# Cardiovascular risk factors differently affect the survival of patients undergoing manual or mechanical resuscitation

**DOI:** 10.1186/s12872-018-0962-6

**Published:** 2018-12-07

**Authors:** Dóra Ujvárosy, Veronika Sebestyén, Tamás Pataki, Tamás Ötvös, István Lőrincz, György Paragh, Zoltán Szabó

**Affiliations:** 1 Department of Emergency Medicine, Faculty of Medicine, University of Debrecen, P.O. Box 19, Nagyerdei krt. 98, Debrecen, 4032 Hungary; 2 Department of Internal Medicine, Division of Metabolism, Faculty of Medicine, University of Debrecen, Debrecen, Hungary

**Keywords:** Resuscitation, Sudden cardiac death, Chest compression

## Abstract

**Background:**

Chest compression is a decisive element of cardio-pulmonary resuscitation (CPR). By applying a mechanical CPR device, compression interruptions can be minimised. We examined the efficiency of manual and device-assisted resuscitation as well as the effects of cardiovascular risk factors on the outcome of resuscitation.

**Methods:**

In our retrospective, randomised 3-year study the data of adult patients suffering non-traumatic, out-of-hospital, sudden cardiac death (SCD) were analysed (*n* = 287). The data were retrieved by processing case reports, Utstein sheets and acute coronary syndrome sheets. We compared the data of patients undergoing manual (*n* = 232) and device-assisted resuscitation (LUCAS-2, *n* = 55). The primary endpoint was the on-site restoration of spontaneous circulation (ROSC).

**Results and conclusion:**

In 37% of the cases ROSC happened. With respect to ROSC an insignificantly more favourable tendency was demonstrated in the case of device-assisted resuscitation (*p* = 0.072). In the Lucas group, a higher success rate occurred even in the case of prolonged resuscitation. We found a better outcome in the Lucas group in the case of CPR started a longer time after the SCD (*p* < 0.05). A positive correlation was established between age and unsuccessful resuscitation (p = < 0.017; *r* = 0.125). An unfavourable correlation was observed between hypertension and the outcome of resuscitation (*p* = 0.018; *r* = 0.143). According to our results the presence of left ventricular hypertrophy poses 5.1-fold risk of unsuccessful CPR (CI: 4.97–5.29).

Advanced age and structural heart diseases can play a role in the genesis of SCD. Importantly, left ventricular hypertrophy and hypertension negatively affect survival.

## Background

There are some 275.000 patients a year in Europe requiring immediate pre-hospital care due to sudden cardiac death (SCD) [1], but regrettably the survival rate is only 10%. [2]. SCD is an unexpected death occurring generally within 1 h of onset of symptoms [3]. The most common cardiovascular diseases that lead to SCD are coronary atherosclerosis and dilated cardiomyopathy, where the loss of cardiac mechanical function and decreased cardiac output can result in the genesis of malignant ventricular arrhythmias [3]. Koldobskiy et al. have found that renal disease, immunosuppression and obesity are associated with lower success rate to cardiopulmonary resuscitation [4]. In another study by Herlitz et al. data of 33,453 patients with out of hospital cardiac arrest were analysed. Initial heart rhythm, non-professional CPR and age were found to strongly affect the outcome of survival [5].

Research findings in the past few years have confirmed that one of the most important elements of successful resuscitation is continuous high-quality chest compression interrupted for the shortest possible times [6, 7]. The early detection of circulatory collapse, cardiopulmonary resuscitation started in time and applied efficiently, early defibrillation and effective post-resuscitation care all serve enhancing the chance of survival and reducing the development of complications [8].

During manual resuscitation interruptions can appear and the depth of compression may also diminish within a short period of time [9, 10]**.** Therefore, manual application of chest compression is often not effective enough. Recently there has been growing need for the development of special devices with the help of which the quality conditions for the protocol requirements can be met. LUCAS-2 (Lund University Cardiopulmonary Assist System) is the most commonly used mechanical chest compression device in the pre-hospital emergency setting. It has been shown to be able to maintain a quality chest compression at the rate of 102 per minute, and at the depth between 5 and 6 cm as it is recommended by the European Resuscitation Council (ERC) [11]. In Hungary, mostly Advanced Life Support (ALS) crews of the National Ambulance Service begin professional CPR on field, sometimes with the additional use of an external chest compression device [12].

In this study we aimed to analyse, in three years’ retrospect, the outcome of manual and mechanical device-assisted resuscitation applied on adult patients suffering sudden cardiac arrest out of hospital, as well as the effects of the risk factors leading to sudden cardiac arrest on survival.

## Methods

Our study was conducted by the Department of Emergency Medicine, University of Debrecen, Hungary. Analysing the period of 01.10.2010–31.12.2013, we processed the data of 287 patients requiring care for sudden, non-traumatic out-of-hospital death. In our retrospective, randomised study we assessed ambulance service case descriptions, Utstein sheets, acute coronary syndrome sheets recorded in the course of treatment, as well as documents outlining previous illnesses, from an electronic hospital database (Medsol). According to the resuscitation method (manual vs. performed with an external chest compression device – Lucas-2), patients were put into two study groups. The primary endpoint was on-site restoration of spontaneous circulation (ROSC). We examined the correlations between the time that passed before resuscitation, initial heart rate, the efficiency of non-professional resuscitation and the outcome. We aimed to determine the neurological status of the patients undergone successful resuscitation when released from hospital, as well as the influence of risk factors leading to SCD on the outcome of resuscitation. The study protocol was approved by the Ethics Committee of the University of Debrecen (number of ethics approval: 16871–2016/EKU 0364/16). The statistical analysis was performed with SPSS for Windows version 24.0. (Armonk, NY: IBM Corp., Released 2016). Variables were characterised with descriptive statistics (case number, average, median and quartile). The differences between the variables were compared in the case of normal distribution by 2-sample t-tests; in the case of non-normal distribution by using Mann-Whitney tests. Where the variances of the examined parameters were significantly different, we applied the Welch correction. The correlation between the variables was established using Spearman rank test. The Odds ratios were calculated with logistic regression. The borderline of significance was *p* < 0.05. In case of Odds ratios 95% confidence intervals were determined.

## Results

**Fig. 1:**
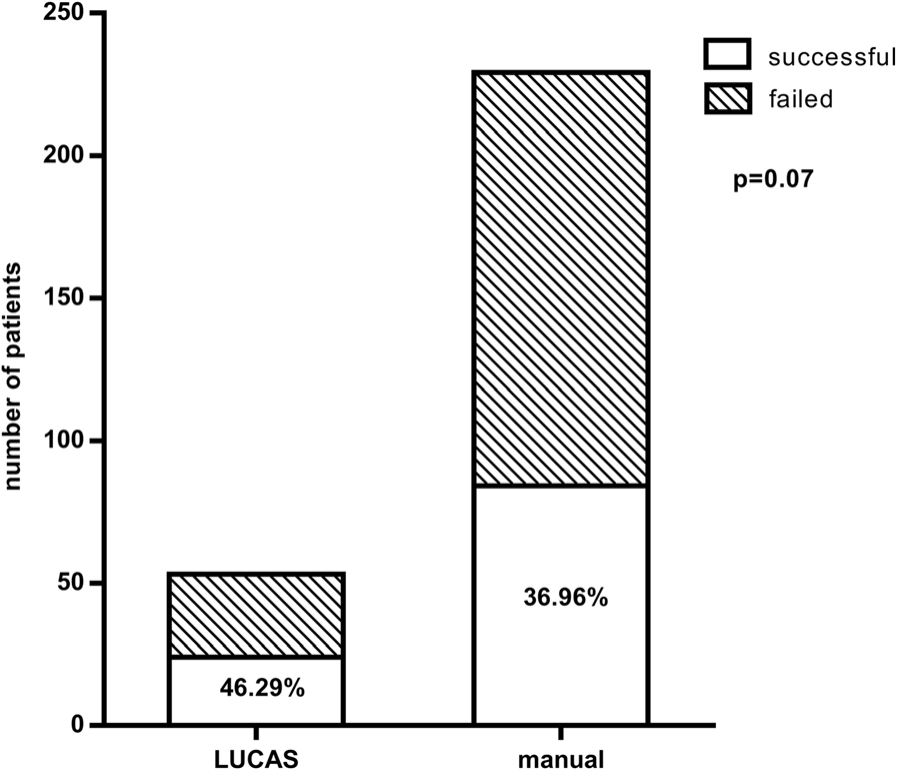
Comparison of the manual and the Lucas group to the return of spontaneous circulation (ROSC). A non-significant tendency is shown implying that Lucas was more efficient

**Fig. 2:**
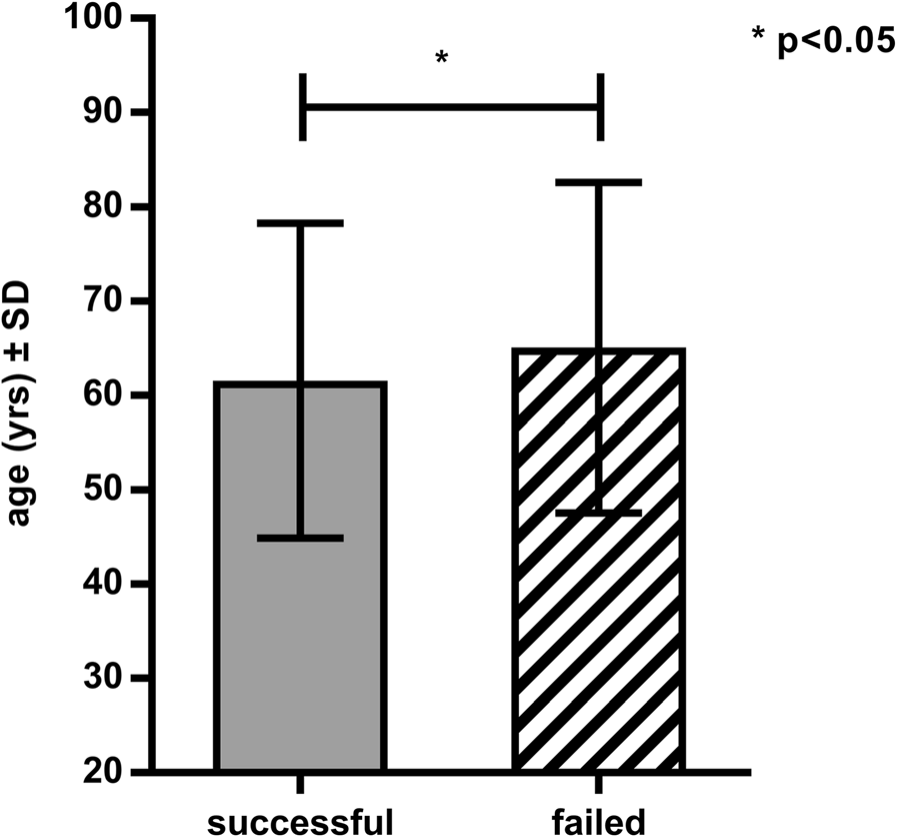
The distribution of age regarding successful and failed resuscitations. Failed resuscitations occurred significantly more frequently in the case of older people

Our patients’ clinical data are summarized in Table 1. In 55 cases, resuscitation was performed using a LUCAS-2 device (19%), while in 232 cases (81%) traditional manual chest compression was applied. One hundred and seven cases of resuscitation (37%) were successful (on-site ROSC, sustained circulation, admission to hospital). Out of the 55 cases of care provided with a mechanical device 26 (46%) were successful, while in the manual group spontaneous circulation returned in 83 cases out of 232 (36%). As regards the patients with successful resuscitation, in 26 cases (23%) a Lucas-2 device was used, while in 83 cases (77%) emergency care was performed manually. Although no significant difference was detected between the two groups with reference to a successful outcome, a non-significant trend implying the efficiency of the Lucas device was observable (*p* = 0.072) (Fig. 1). The mean age of the successfully resuscitated group was 64 ± 13 years, while it was significantly higher (65 ± 13 years) in the case of the deceased (Fig. 2). A significant positive correlation was observed regarding older age and unsuccessful resuscitation (p = < 0.017; *r* = 0.1246). In the case of both men and women 39% of resuscitation attempts resulted in ROSC.

**Table 1:** Clinical data of the study population. The patients were categorized into two groups based on the method of care received

Parameter	Manual	Device-assisted
Case number (items)	232/287 (80%)	55/287 (20%)
Age (years)	61 ± 16.98	65 ± 17.54
Male	244 (67%)	113 (72%)
Female	120 (33%)	43 (28%)
Resuscitation period (minutes)	38 ± 2.51	49 ± 1.84

**Fig. 3:**
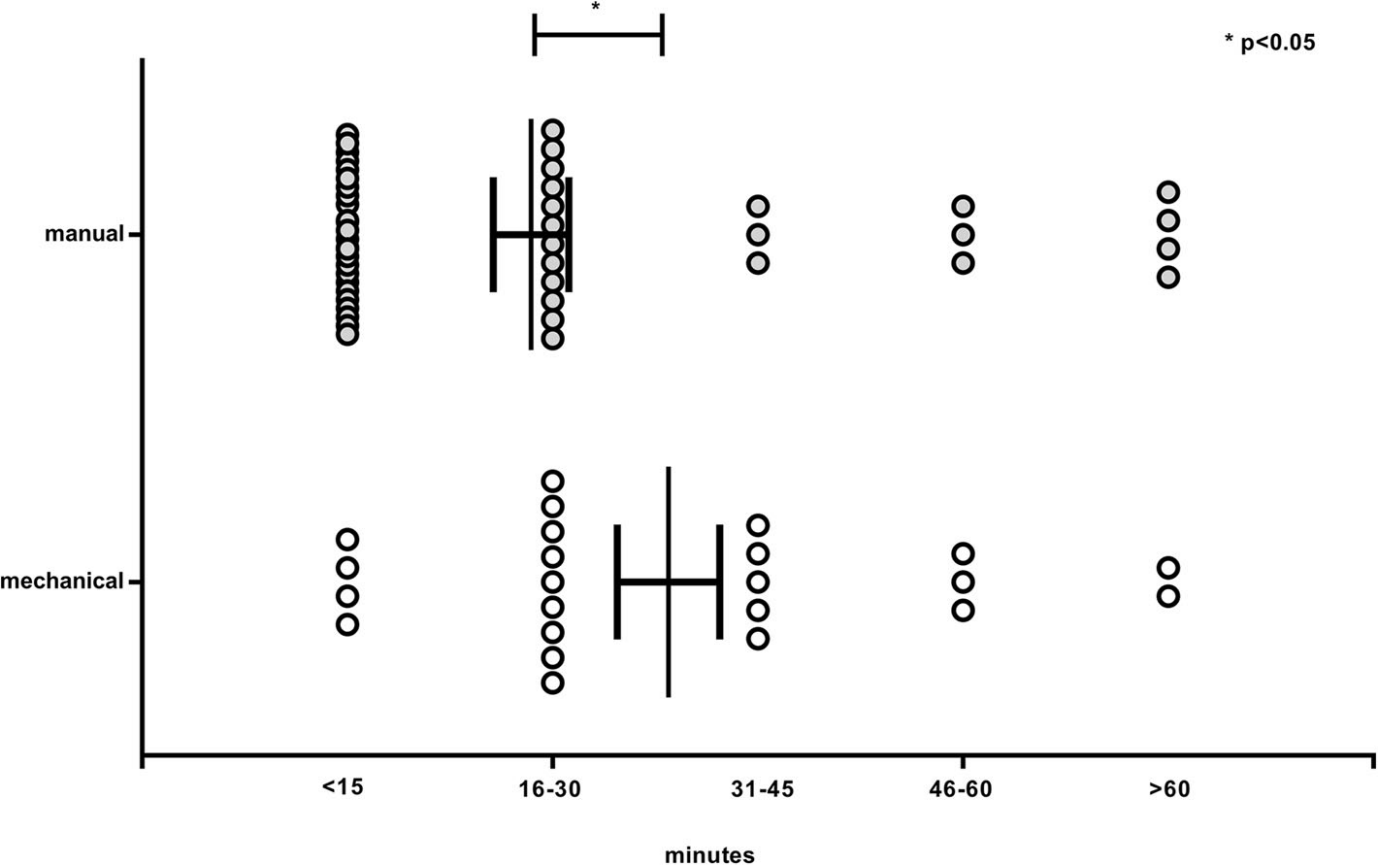
The time that elapsed before return of spontaneous circulation (ROSC)

**Fig. 4:**
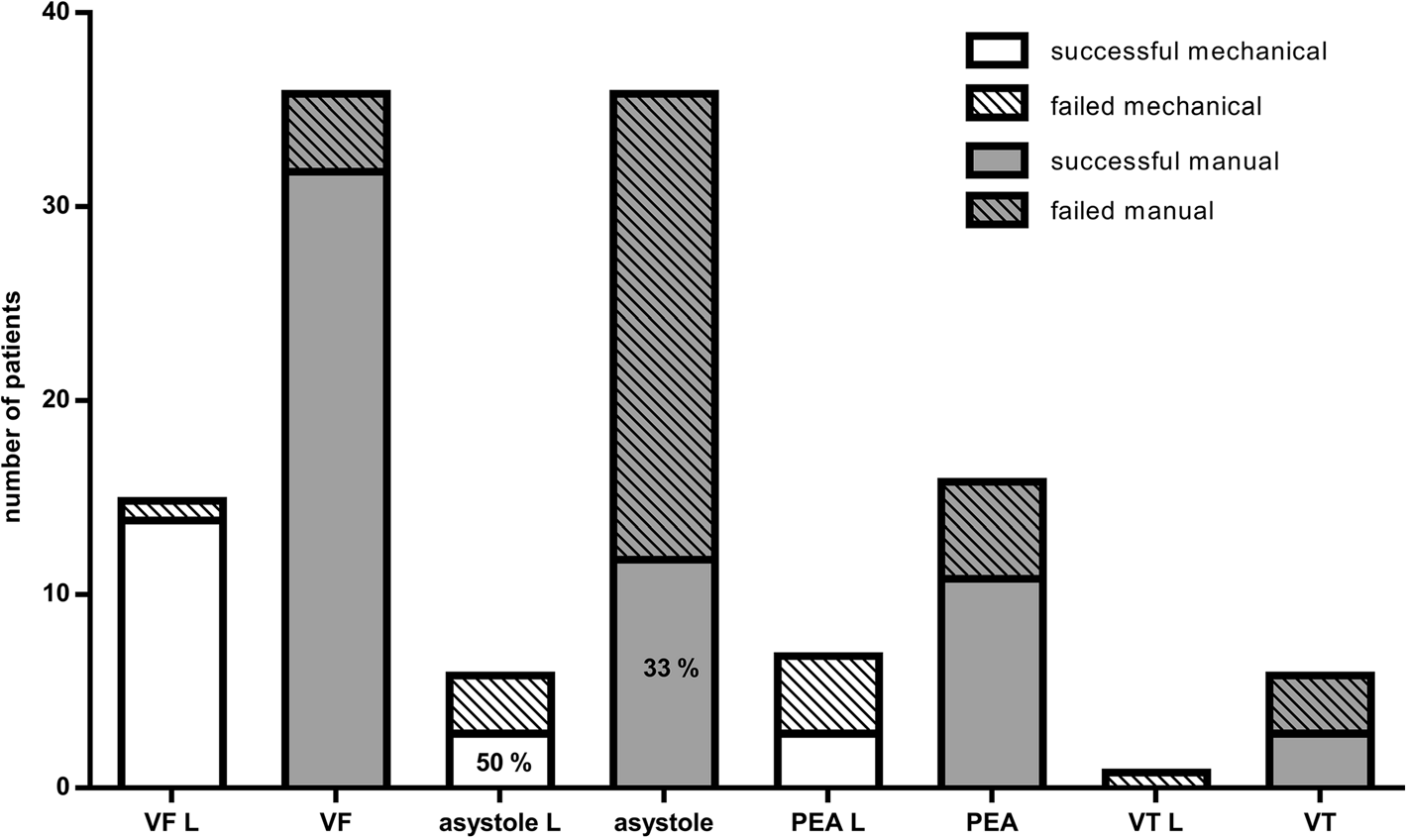
Connection between first observed rhythm and ratio of return of spontaneous circulation (ROSC). Ventricular fibrillation is a favourable indicator regarding the outcome of resuscitation in both Lucas and manual groups. In the cases of initial asystole, resuscitation by using a Lucas device was significantly more effective. VF: ventricular fibrillation, L: Lucas, PEA: pulseless electrical activity, VT: ventricular tachycardia

In 72 cases we managed to collect information on the length of time that elapsed before resuscitation was started and the time elapsed until the ROSC happened. In 31 cases (43%) ROSC returned within 1–15 min, in 4 cases of which (13%) this happened by using a Lucas-device and in 27 cases (87%) by manual resuscitation. In 21 cases (29%) ROSC occurred within 16–30 min, with a ratio of 12 manual to 9 LUCAS resuscitations (57 and 43% respectively). In the case of eight patients (11%) ROSC was observed within 31–45 min, in 5 cases (71%) in the Lucas-2 group and in 3 cases (29%) in the manual group. In six cases (8%) spontaneous circulation returned in 46–60 min, in 3 cases in both categories (50–50%). Finally, regarding 6 patients (8%) spontaneous circulation returned after more than 1 h of resuscitation, in 2 cases (40%) by the use of Lucas-2 and in 4 occasions (60%) during manually performed CPR. In the Lucas group there was a higher rate of success even in the case of prolonged resuscitation (*p* < 0.05) **(**Fig. 3**).** Correlation between the first documented rhythm and the success of resuscitation were also examined. Registration of the first rhythm was available in the case of 127 patients: in 42 cases (33%) asystole, in 52 cases (41%) ventricular fibrillation, in 5 cases (4%) ventricular tachycardia, in 21 cases (16.5%) pulseless electrical activity (PEA) and in 7 cases (5.5%) other rhythms were described. Regarding asystole, resuscitation was successful in 14 cases (33%), 3 of which (21%) occurred by using a Lucas-2 device. With reference to the 52 ventricular fibrillation cases spontaneous circulation returned in 47 cases (90%), 14 of which (30%) occurred by using a mechanical device. In the 21 cases of PEA, emergency care was successful on 13 occasions (62%). In the cases of the initial asystole, resuscitation by using a Lucas device was significantly more effective **(**Fig. 4**).**

We found non-professional resuscitation documented in eighty cases; on 34 occasions (42.5%) resuscitation was started by an untrained individual, while in 46 cases (57.5%) initial care was provided by the ambulance service. Neurological status of patients (based on Cerebral Performance Category - CPC criteria) determined at the time of release from hospital was also established [13]. There were data available on the neurological status of 70 manually resuscitated patients. In twenty cases (28.5%) we awarded 1 score on the CPC scale, in 5 cases (7%) 3 scores, in 4 cases (6%) 4 scores and in 41 cases (58.5%) 5 scores to the neurological status of the patient. We found data on the neurological status of twenty-one patients successfully resuscitated using a Lucas-2 device in the database. In two cases (9.5%) we awarded 1 score to the patients’ neurological status, in the case of 1 patient (5%) 4 scores were awarded, and in 18 cases (85.5%) 5 scores were awarded. Regarding neurological end status we set up two categories: patients in the categories CPC 1–2 scores were qualified as “good” and patients with CPC 3–4-5 scores were qualified as “bad” [14]. In the case of successful resuscitations, neurological end status was better in the manually resuscitated group (*p* < 0.05). In the case of one resuscitation performed with a chest compression device, we found a remark about a chest injury in the pathological findings.

In one hundred and sixty-seven cases (87%) hypertension was confirmed (with RR permanently above 140/90 mmHg based on earlier documentation). In the cases of hypertension, resuscitation failed in 104 cases (62%) and it was successful in 63 occasions (38%). Significant correlation was observed between hypertension and the negative outcome of resuscitation (*p* = 0.018; *r* = 0.143). According to our calculations the presence of hypertension poses 1.82-fold risk of unsuccessful resuscitation. There were data available on patients’ lipid parameters in 149 cases. Fifty-four percent of patients had hypercholesterolaemia (serum cholesterol> 5.2 mmol/L), while in 46% no lipid abnormalities were evaluated. In the case of higher serum cholesterol resuscitation failed in 37 cases (46%) and was successful in 33 occasions (54%). Considering these observations we found no significant correlation between hypercholesterolaemia and the outcome of resuscitation (*p* = 0.379; *r* = 0.024).

In 174 cases we found information on ventricular hypertrophy (LVH) (interventricular septal and/or posterior wall thickness > 12 mm). In 151 cases (87%) LVH was present while in 23 subjects (13%) this was not confirmed. In the cases of existing LVH resuscitation failed in 109 cases (72%), while in 42 occassions (28%) it resulted in ROSC. We found a significant correlation between left ventricular hypertrophy and the unsuccessful resuscitation (*p* = 0.0009; *r* = 0.1995). Importantly, according to our calculations the presence of LVH poses 5.1-fold risk of failed resuscitation **(**Table 2**.).**

**Table 2:** Correlations between riskx factors and the return of spontaneous circulation (ROSC) and the parameters negatively affecting the effectiveness of resuscitation

Parameters	*r* value	*p* value
Taking of oral anticoagulants	−0.018	0.880
Previous stroke	0.010	0.440
Previous myocardial infarction	0.007	0.458
Obesity	−0.043	0.277
Type I. diabetes mellitus	−0.050	0.242
Type II. diabetes mellitus	−0.023	0.381
Left ventricular hypertrophy	0.200	0.001
Hypertension	0.143	0.018
Hyperlipidaemia	−0.024	0.379
Odds ratios in case of parameters negatively affect ROSC		
Parameters	OR	CI 95%
Left ventricular hypertrophy	5.131	4.965–5.293
Hypertension	1.822	1.748–1.891

*r* value: correlation coefficient, *p* value: level of significance, *OR*: Odds ratio quantifying how strongly the presence of the comorbid factors are associated with the negative outcome of resuscitation, CI: 95% confidence interval. If the interval not contains 1 the finding is significant

In 193 cases data on body mass index (BMI) were detected. In 78 cases (40%) obesity (BMI > 25) was confirmed, while in 115 cases (60%) no pathological overweight was observed. In the cases of obesity, circulation did not return in 44 patients (56%), while regarding 34 subjects (44%) resuscitation was successful. Therefore no significant correlation could be established between body weight and the outcome of resuscitation (*p* = 0.2766; *r* = 0.043).

In 211 cases data on any previous myocardial infarction were found. In 56 cases (26.5%) earlier heart attacks were proven, while in the case of 155 patients (73.5%) there were no information indicating any previous myocardial infarction. As regards post-infarction patients, resuscitation was unsuccessful in 35 (62.5%), and successful in 21 cases (37.5%). Thus no significant correlation was found between earlier myocardial infarction and the outcome of resuscitation (*p* = 0.4579; *r* = 0.007).

Sixty-six patients had documented diabetes mellitus (36%), while in 118 cases (64%) there were no data indicating the disease. No significant correlation has been established between diabetes mellitus and the success of resuscitation.

Out of 192 patients we found data on earlier treatment for stroke in 13 cases (7%). As regards the patients treated for stroke, resuscitation failed in 8 cases (61.5%), while spontaneous circulation returned in 5 cases (38.5%). Consequently no significant correlation could be established between earlier stroke and the outcome of resuscitation (*p* = 0.4399; *r* = 0.0104).

In ninety-three cases there were data indicating that the patients took anticoagulants. Cardiopulmonary resuscitation (CPR) was successful in the case of 45 patients and failed in 48 subjects. Based on these data, no significant correlation between anticoagulant therapy and successful ROSC could be established (*p* = 0.8798; *r* = 0.0176).

## Discussion

In the past twenty years cardiovascular mortality has fallen as a result of preventive measures, although cardiovascular diseases continue to claim the lives of 17 million people a year all over the world, 25% of which are casued by SCD [15]. Recent studies have shown that the success of resuscitation highly depends on quality, continuous, and non- interrupted chest compressions [7, 11]. Previously it has also been proven, that during resuscitation the quality of chest compressions are significantly better by LUCAS-2 device compared to manual CPR, because the mechanical device may reduce the no-flow fraction (NFF) [11, 16]. Using mechanical compression higher coronary and cerebral perfusion pressure can be sustained, which is clearly determinative for both ROSC and neurological outcome [17, 18]. The use of a mechanical device allows the emergency medical service (EMS) crew to carry out continuous quality resuscitation during transport or even in confined spaces [2].

In our present study we intended to examine the outcome of manual and mechanical device-assisted resuscitation in patients suffering sudden cardiac arrest out of hospital, as well as the effects of the risk factors leading to sudden cardiac arrest on survival. Advanced age, left ventricular hypertrophy, and hypertension have been found to be the most adversely influencing substrates of resuscitation. According to our important observation, left ventricular hypertrophy poses 5.1-fold risk, while the presence of hypertension means 1.82-fold risk of unsuccessful resuscitation. Although increased body weight showed no significant correlation with the outcome of resuscitation, our data imply that pathogenic factors playing a role in the thickening of the left ventricular wall, including obesity, may indirectly be important underlying factors regarding survival.

In previous investigations successful resuscitation has been shown to fall to a great extent if the initial heart rhythm analysis confirmed asystole. In such cases only 10% of patients have been successfully stabilised on the spot moreover up to 2% survived hospital treatment. It has similarly been confirmed as an unfavourable sign for the prognosis if the cardiac arrest occurred due to pulseless electrical activity (PEA) [19]. In our study the initial rhythm felt upon arrival on the site has also clearly affected the outcome of CPR. Only a succes rate of 62% was found in the case of PEA, while the presence of ventricular fibrillation meant a higher chance of 90% for the return of circulation.

In more than 57% of the cases the treatment was started by the ambulance service. Unfortunately, in these particular cases there was no non-professional resuscitation or first aid. Therefore we can conclude that for the majority of our patients a hypoxic and/or anoxic period may have been a considerable risk factor and may have influenced the outcome of resuscitation fundamentally. This is probably the explanation for the unfavorable tendency observed in the neurological status of the resuscitated. The disappointingly low number of non-professional resuscitations draws attention to the importance of teaching basic life support (BLS) and wide-scale awareness raising about the chance enhancing potential of early care.

We underline that the frequency of traumatic injuries in the course of CPR using mechanical compression devices was not higher compared to manual resuscitation, showing the clinical applicability of this method.

Importantly, mechanical resuscitation did not have a worse outcome compared to the manual method, despite the fact that chest compression had to be temporarily interrupted while the compression device was placed on the patient [20].

According to our findings it can be concluded that the use of chest compression devices during resuscitation is safe and effective, and offers a significant help for the emergency care providers. We highlight the importance of preventive medical approach that aims to reduce the frequency of hypertension and ventricular hypertrophy, as significant factors unfavorably affecting survival of patients suffering sudden cardiac death. In our study a relatively low number of non-professional resuscitations have been observed that emphasizes the significance of quality education of basic life support.

## Conclusions

Our research findings highlight the pathogenic role of advanced age and related structural heart diseases leading to SCD, and draw attention to left ventricular hypertrophy and hypertension as most important factors negatively affecting survival. Our data suggest that chest compression devices can provide effective and safe help to emergency care providers. We emphasize that a preventive medical attitude applied in order to prevent myocardial structural and electrical remodelling may reduce the pathogenic factors leading to SCD and thereby contribute to improving mortality statistics.

## Study limitation

During our investigations all available source of information was utilized, nevertheless the exquisite collection of the examined clinical parameters due to obvious reasons was not possible. However statistically reliable amount of data were used during our calculations.

## Abbreviations

BLS: Basic life support
BMI: Body mass index
CI: Confidence interval
CPC: Cerebral performance category
CPR: Cardiopulmonary resuscitation
ERC: European Resuscitaion Council
LVH: Left ventricular hypertrophy
NFF: No-flow fraction = no-flow time/episode duration (time with ROSC)
OR: Odds ratio
PEA: Pulseless electrical activity
ROSC: Return of spontaneous circulation
SCD: Sudden cardiac death
SD: Standard deviation
VF: Ventricular fibrillation
VT: Ventricular tachycardia
